# A Review of the Residual Stress Generation in Metal Additive Manufacturing: Analysis of Cause, Measurement, Effects, and Prevention

**DOI:** 10.3390/mi14071480

**Published:** 2023-07-24

**Authors:** Nabin Bastola, Muhammad P. Jahan, Nithin Rangasamy, Chandra Sekhar Rakurty

**Affiliations:** 1 Department of Mechanical and Manufacturing Engineering, Miami University, Oxford, OH 45056, USA; bastoln@miamioh.edu; 2 The M. K. Morse Company, Canton, OH 44707, USA; rangasamyn@mkmorse.com (N.R.); rakurtys@mkmorse.com (C.S.R.)

**Keywords:** metal additive manufacturing, residual stress, distortion, post-processing

## Abstract

Metal additive manufacturing (AM) is capable of producing complex parts, using a wide range of functional metals that are otherwise very difficult to make and involve multiple manufacturing processes. However, because of the involvement of thermal energy in the fabrication of metallic AM parts, residual stress remains one of the major concerns in metal AM. This residual stress has negative effects on part quality, dimensional accuracy, and part performance. This study aims to carry out a comprehensive review and analysis of different aspects of residual stress, including the causes and mechanisms behind the generation of residual stress during metal AM, the state-of-the-art measurement techniques for measuring residual stress, various factors influencing residual stress, its effect on part quality and performance, and ways of minimizing or overcoming residual stress in metal AM parts. Residual stress formation mechanisms vary, based on the layer-by-layer deposition mechanism of the 3D printing process. For example, the residual stress formation for wire-arc additive manufacturing is different from that of selective laser sintering, direct energy deposition, and powder bed fusion processes. Residual stress formation mechanisms also vary based on the scale (i.e., macro, micro, etc.) at which the printing is performed. In addition, there are correlations between printing parameters and the formation of residual stress. For example, the printing direction, layer thickness, internal structure, etc., influence both the formation mechanism and quantitative values of residual stress. The major effect residual stress has on the quality of a printed part is in the distortion of the part. In addition, the dimensional accuracy, surface finish, and fatigue performance of printed parts are influenced by residual stress. This review paper provides a qualitative and quantitative analysis of the formation, distribution, and evolution of residual stress for different metal AM processes. This paper also discusses and analyzes both in situ and ex situ measurement techniques for measuring residual stress. Microstructural evolution and its effect on the formation of residual stress are analyzed. Various pre- and post-processing techniques used to countermeasure residual stress are discussed in detail. Finally, this study aims to present both a qualitative and quantitative analysis of the existing data and techniques in the literature related to residual stress, as well as to provide a critical analysis and guidelines for future research directions, to prevent or overcome residual stress formation in metal AM processes.

## 1. Introduction

Additive manufacturing (AM) or three-dimensional (3D) printing is a type of manufacturing technology in which feedstock materials are joined in layer-by-layer fashion to create parts from 3D CAD data [1,2]. AM enables fabrication of parts with complex geometries with a relatively short cycle time compared to traditional manufacturing processes or subtractive manufacturing. The parts fabricated with 3D printing process (rapid prototyping) are suitable for making prototypes for scientific analysis and assembling final parts, which can be used in wide-range of engineering applications, including automobile, aerospace, and medical [3,4,5]. The major advantages of AM process over subtractive manufacturing include rapid prototyping, a relatively short cycle time, reduction in material waste, fabrication of complex shaped geometries, high flexibility, increased functionality, and fabrication of lightweight parts, which is simply impossible to achieve via traditional manufacturing processes [6,7].

**Figure 1:**
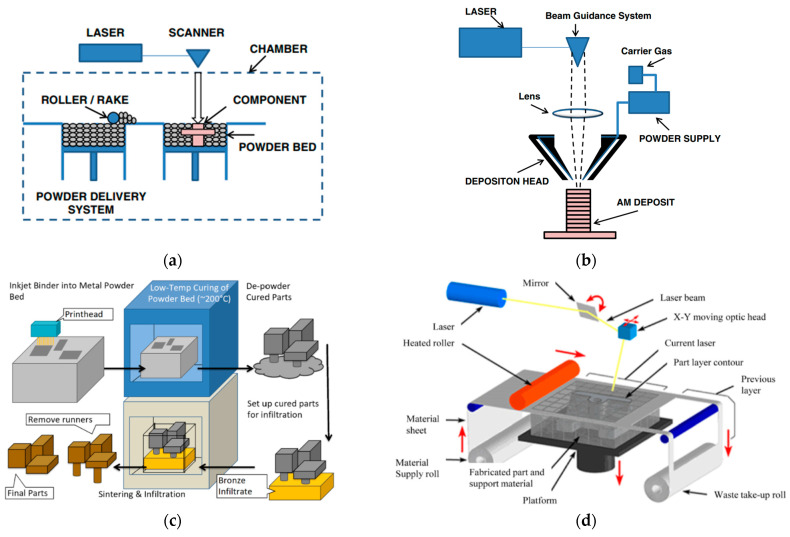
Illustration of AM technologies. (**a**) Powder bed fusion (PBF) [9], (**b**) direct energy deposition (DEP) powder-based [9], (**c**) binder jetting [11], and (**d**) laminated object manufacturing (LOM); red arrows represent the motion of X-Y moving optic head, material sheet, platform, heated roller, and the mirror [12]. (With kind permission from Elsevier and Springer Nature).

The classification of AM technologies for metal 3D printing uses their heat source and feedstock. Powder bed fusion (PBF) and directed energy deposition (DED) are the two primary categories of AM technologies. PBF includes AM techniques that use a bed of powder particles. A roller or rake is used to spread a thin layer of powder across the baseplate, and an electron beam or laser is used to scan the layer geometry, which melts the powder particles into a solid structured layer [8]. Selective laser sintering (SLS) is one of the main examples of PBF 3D printing technology. Various types of material, including metals, polymers, composites, and ceramics, can be used for the SLS manufacturing process. PBF technologies are typically renowned for their high accuracy, better surface quality, and fast printing. The mechanical quality of SLS parts is typically rated higher than parts manufactured via extrusion or injection molding. A schematic diagram of a PBF process is presented in Figure 1a [9]. The DED process involves using either a powder or wire feedstock. In a DED process, a powder or wire is passed through nozzles and then encounter a heat source (laser), which directly melts the material [10]. The DED process is commonly used for repairing parts or adding more materials to existing parts. Typically, metals and alloys are used in the form of wire or powder to form objects; however, ceramics and polymers can also be utilized. A schematic diagram of the DED process is illustrated in Figure 1b [9].

**Figure 2:**
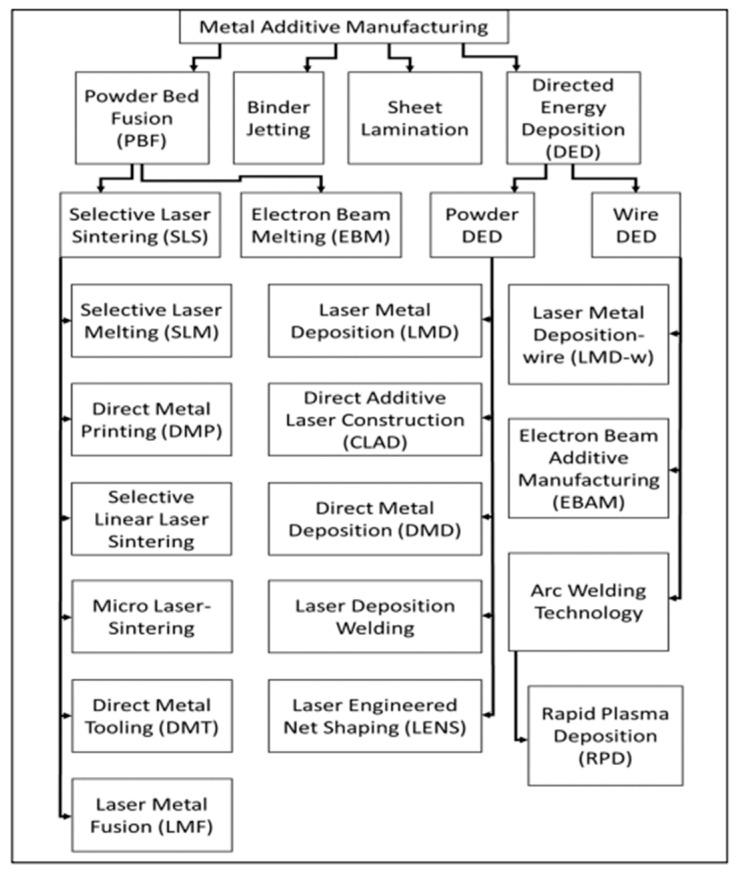
Classification of AM technologies for metals [13]. (open access).

In addition to PBF and DED technologies, binder jetting and sheet lamentation are also utilized for metal additive manufacturing. Binder jetting utilizes a polymeric liquid (binder) to bond layers of powder material into the desired layer shape. The main advantage of binder jetting over other AM technologies is the compatibility of the process with any powdered material. The shaping process in binder jetting is completed at room temperature, which reduces issues of oxidation, phase change, and formation of residual stress. The main disadvantage of binder jetting is the requirement for post-processing, such as curing and densification. A schematic diagram of binder jetting is presented in Figure 1c [11]. Sheet lamentation is another type of AM technology, in which sheets of material are bonded together using a thermoplastic powder under the application of compression and heat. Laminated object manufacturing (LOM) is the most prominent example of sheet lamentation technology. The major advantage of sheet lamentation technology is the ability to fabricate mechanically stronger parts compared to traditional manufacturing processes. A schematic diagram of laminated object manufacturing (LOM) is presented In Figure 1d [12]. The classification of various AM technologies for metal 3D printing is presented in Figure 2 [13].

Fast-paced research and development in metal-additive manufacturing, which uses powder metals ranging from steel, aluminum, and titanium alloys, to Ni-based super alloys for high temperatures, has been a major factor in the increasing acceptance of AM in recent years. Despite the wide range of applications of AM technology in various scientific fields, wider adoption of these technologies is limited, due to challenges associated with the design and manufacturing of parts. The high-intensity laser beam used to melt the metal materials can locally increase the temperature distribution of the metal to thousands of degrees. The laser melting in the AM process leads to the formation of high residual stress in parts fabricated through this process compared to in the traditional manufacturing process [14]. Similarly, the surface quality of fabricated AM parts is inferior compared to subtracting processes, because of the process involving the layer-by-layer deposition of metal [15]. Additionally, for systems based on metal powder, in addition to the stair-step effect, there is aggregation of partially melted particles, which causes surface degradation. Dimensional accuracy can also be negatively impacted by warping, shrinkage, expansion, thermal stresses, and other factors. In addition to a poor appearance, AM parts also exhibit low fatigue resistance, due to an inferior surface quality [16,17].

Among the various challenges associated with AM of metals, residual stress and part distortion have been the major obstacles encountered by researchers. These two factors are particularly important to resolve, because of their direct association with the dimensional accuracy and mechanical performance of the fabricated parts [18]. While this paper is concentrated on a literature review of residual stress and distortion in metal additive manufacturing, selective dealloying of alloy materials (magnesium alloys, aluminum alloys) manufactured via AM technologies is also an important obstacle to overcome. The selective corrosion of alloy materials results in the dissolution of certain elements from the alloy, leaving behind a porous material with lower mechanical strength [19]. Corrosive contaminants can negatively impact both the environment and human health if ingested. Therefore, careful measures should be taken when manufacturing alloy parts for various engineering applications.

**Figure 3:**
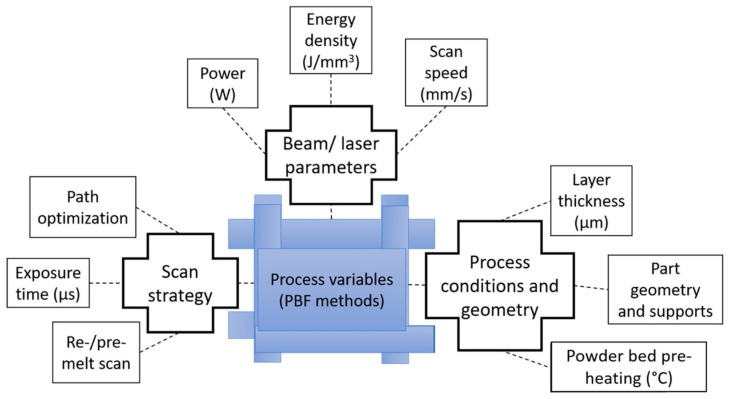
Process variables (PBF methods) affecting the residual stress characteristics in AM parts [21]. (With kind permission from Elsevier).

Residual stress in AM metals directly affects the part quality and performance under various mechanical loadings and applications, resulting in part failure. Residual stress in metal parts has the tendency to either pull or push the materials and deform the parts, depending on the direction of residual stress. As a result, AM metal parts are susceptible to distortion in the presence of residual stress. The structural reliability of a part under cyclic loading is negatively impacted by residual stress, in terms of the performance of the part. The characteristics of the residual stress (size, distribution, and direction) depend, not only on the features of AM technologies, such as the heat source, material feedstocks, and the ambient conditions, but also on the input process parameters, including the scanning speed, laser power, scanning strategy, and build orientation [20]. The process variables affecting the residual stress of manufactured parts are presented in Figure 3 [21].

Rapid heating, cooling, and melt-back, which involves simultaneously melting the top material layer and remelting the lower, previously hardened layers, are characteristics of the special thermal cycle of metal AM. For additive manufacturing of alloy elements such as magnesium alloys or aluminum alloys, in addition to rapid cooling, which acts as the transformation kinetic, selective dealloying can influence the nucleation of the new phase. Additive manufacturing of Mg powder is difficult, because it readily oxidizes in air under the influence of heat. Thus, alloying is conducted by adding elements such as calcium, zinc, and manganese, in order to slow the degradation. The addition of these elements affects the integrity, grain structure, and heat resistance of manufactured components. The low presence of alloying elements in magnesium powder can result in a loss of relative density in the deposit, whereas increasing the content of alloying elements can result in micro-cracks in a deposit [22]. Due to the severe residual stress gradients that result from the particular heat cycle in metal additive manufacturing, component distortion is a major concern [23,24]. To lessen residual stress and part deformation in metal AM, several techniques have been investigated [23]. The most common method for reducing residual stress is to lower the temperature gradient by preheating the substrate or feedstock material [25,26,27].

A wide range of difficulties related to the metal additive manufacturing process have been the subject of numerous published or continuing investigations, including processability, functionality, and surface integrity. The issue of residual stress in the AM process has gained much importance in recent years, as AM processes are finding industrial applications. Therefore, this study aims to provide a comprehensive overview of various aspects of residual stress generation in AM, including the mechanism, cause, effect, measurement techniques, and post-processing techniques used to alleviate the negative effects of residual stress on part quality and application.

## 2. Types and Mechanisms of Residual Stress in AM

Residual stresses (RS) are classified based on different length scales. Depending on the length, the residual stress can be type I, type II, or type III [28]. Type I residual stress occurs at a macro level. Macro RS deform the parts if the boundary conditions are changed. Type II residual stress occurs at the grain scale of the material. Type II residual stress exists due to the anisotropy of the material and varying grain orientation. Type III residual stress occurs at the nano-level. The existence of type III residual stress is explained by the defects present in the parts at the atomic level, such as dislocation, porosities, and impurities. Type I residual stresses are formed due to the non-uniform plastic deformation induced by the thermal and mechanical loads applied during the manufacturing process. As a result, Type I residual stress is generated during every stage of the manufacturing process, where uneven thermal and mechanical loading is frequently applied to manufacture the desired components. Type II and Type III residual stresses are always present in polycrystalline materials, due to the presence of microscopic defects, impurities, and property differences due to different grain orientations. In regards to the manufacturing of AM metal components, Type I residual stress has been studied the most In the literature. The fatigue properties of AM metal parts are directly affected by the presence of macroscopic and anisotropic Type I residual stress. Type I residual stress changes noticeably when the dependent processing parameters and ambient conditions of the AM process are changed. In addition, the measurement of Type II and Type III residual stresses is difficult compared to macroscale residual stress. Therefore, researchers focus more on macroscale residual stress formation in metals, because its effects on part quality are apparent and can be determined through visible distortion and cracks on the surface of the parts [29,30].

The formation of RS In metal AM Is Induced by the Input thermal energy, which creates a differential heating of solid and differential cooling during the solidification process. Contraction tensile stresses are generated during the melting process, due to the change in volume. Similarly, tensile stresses are generated during the solidification process of AM. In some metals and alloys, phase transformation causes volume change during the melting process, resulting in the formation of tensile stresses [29]. Non-uniform thermal contractions and expansions of surrounding materials near the heat-affected zone are induced by temperature gradients, which results in the formation of additional stresses [31]. In AM process such as DED, SLM, and LM, operating at room temperature, the residual stress tends to be higher compared to that generated during the EBM process, which operates at higher temperatures. The cooling rate in the EBM process is significantly lower compared to DED and SLM processes. This is accomplished by preheating the substrate using a primary heat source, such as an electron beam. Under high operating temperatures, the thermal gradient between the melting temperature and powder bed temperature is reduced, resulting in lower residual stress [32,33]. In direct additive manufacturing (DAM) processes, such as SLM and EBM, the residual stress and distortion is mainly affected by the subsequent high-temperature debinding and sintering process, resulting in volume shrinkage. In the DAM process, thermal residual stresses are developed due to the temperature gradient of the melt pool and the ambient condition. Contraction stress is developed during and after the solidification and cooling process. Similarly, constraint stress is developed due to the displacement constraints in the solidified region. In indirect additive manufacturing processes such as SLA, thermal stress is developed due to the temperature gradient, and contraction stress is developed due to solidification and cooling [34].

**Figure 4:**
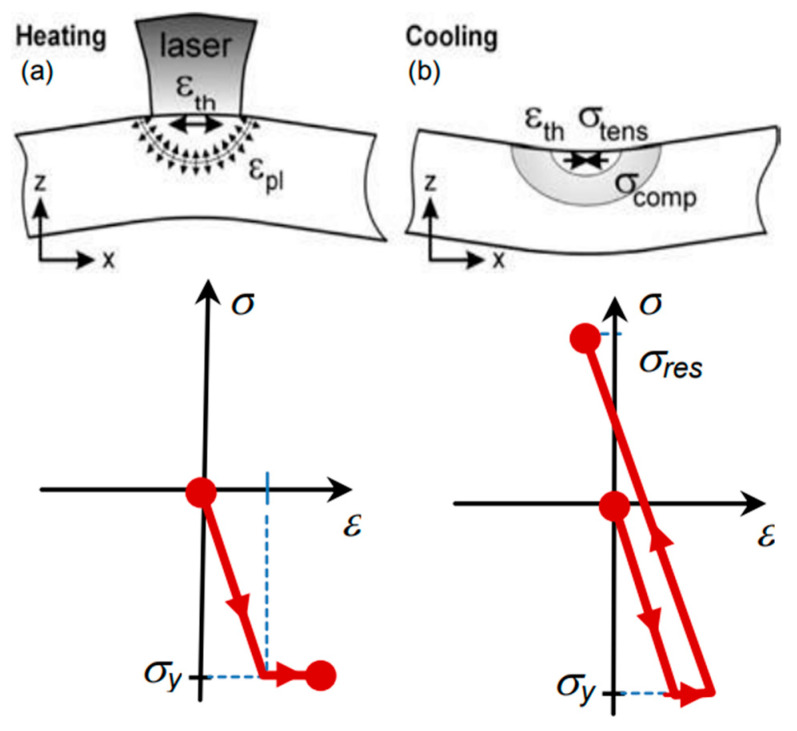
Temperature gradient mechanism (TGM) model. (**a**) Localized deformation during the heating-phase and (**b**) cooling-phase in the heat-applied zone; red arrows show the direction of residual stress formed during heating phase (compressive RS) and cooling-phase (tensile RS) [29]. (Open access).

Mercelis et al. [24] explained the formation mechanism of residual stress with the help of a temperature gradient mechanism (TGM) model and cool-down phase model. The temperature gradient mechanism (TGM) model describes residual stress formation in the AM process, involving unique thermal cycles of melting, solidification, and remelting. The TGM model explains that the high-intensity heat source in the AM process elevates the material temperature locally In the heat-applied zone with respect to the nearby surrounding materials. The localized heated zone tends to expand due to the application of high-intensity energy sources; however, this expansion is restricted by the surrounding materials that are cooler and expand less. Hence, compressive stress is developed in the heat-applied zone. When the heated zone is left to cool, its contraction process is again restricted by the surrounding materials. As a result, permanent tensile residual stresses are developed in the heat-treated zone. The temperature gradient mechanism model is presented in Figure 4 [29].

The second mechanism that explains the formation of residual stress in AM processes is the cool-down phase mechanism. AM processes are characterized by the layer-by-layer deposition of materials. A recently deposited layer tends to shrink during the cooling process; however, the shrinkage process is restricted by the layer below the recently added layer. This results in the formation of tensile stress in the newly deposited later, and the lower layer develops compressive stress. When a material is deposited layer-by-layer, compressive stress is continuously developed in the lower layer. In order to create a moment equilibrium, the bottom layer of the part develops a tensile stress zone, forming a tensile–compressive–tensile stress distribution from the top to the bottom of the part.

## 3. Effects of Residual Stress on Part Quality and Mechanical Characteristics

**Figure 5:**
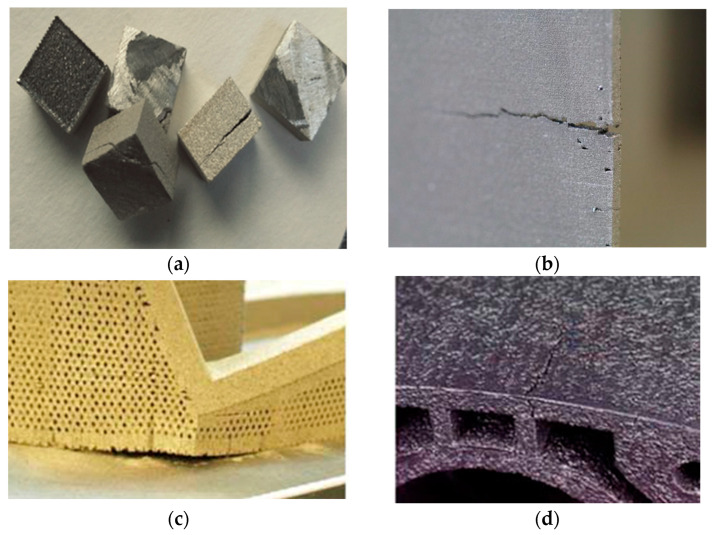
(**a**) Cracking of an Inconel-718 alloy part [36], (**b**) cracking of a Ti-6Al-4V alloy part [37], (**c**) distortion and separation from the base plate [26], and (**d**) crack formation [26] (with kind permission from Elsevier and Springer Nature).

**Figure 6:**
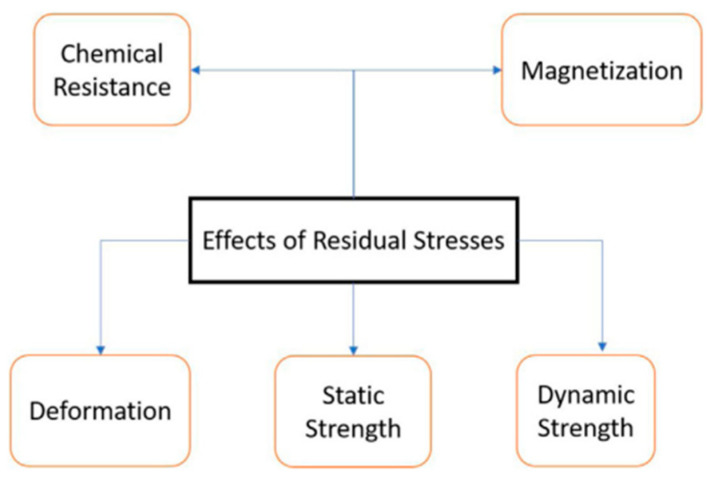
Effects of residual stress on material properties [21] (with kind permission from Elsevier).

The fatigue life of fabricated metal parts is reduced in the presence of tensile residual stresses, because they act as a driving force for the formation and propagation of cracks under cyclic loading [35]. Figure 5a,b [36,37] show the cracking of SLM parts due to the non-uniform residual stress accumulated during the fabrication process. Figure 5c [26] shows the distortion and separation of a part from the base plate due to presence of residual stress, and Figure 5d [26] shows the crack formation in the presence of RS under the Influence of loading. Another common failure for thin-wall parts is structural warping due to RS during fabrication without an adjacent base. In addition to limiting the fatigue life, the dimensional accuracy of parts is reduced in the presence of residual stress, which can lead to a poor fit of parts during the assembly process [38]. Material properties such as chemical resistance, magnetization, deformation, warpage, static strength, and dynamic strength are lowered by tensile residual stresses. The effect of residual stress in fabricated AM part properties is presented in Figure 6 [21].

AM technologies lack product quality control compared to subtractive manufacturing processes, because of the undesired distortion and fatigue-induced cracking caused by the formation of RS during manufacturing; however, it should be emphasized that a material’s performance is not always harmed by residual stress. For example, shot peening introduces residual compressive stress on the surface of parts to enhance fatigue performance [39]. However, RS severely impacts the quality degradation of AM products and entails possible threats to the mechanical performance of parts, limiting the further implementation of AM technologies at a wider scale.

## 4. Measurement of Residual Stress

**Figure 7:**
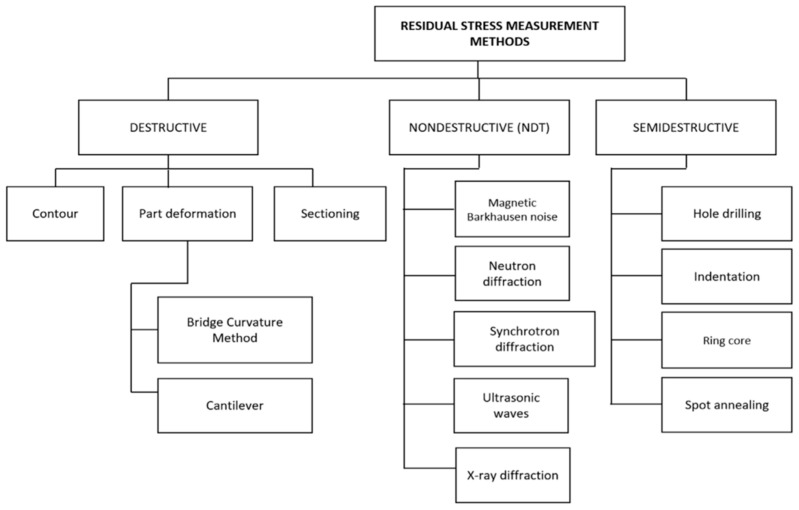
Measurement techniques for residual stress in metals [21] (with kind permission from Elsevier).

Measurement methods for residual stress are classified into three major groups, depending on whether the sample is destroyed or not. Based on the destruction of samples, measurement techniques are categorized as non-destructive, destructive, and semi-destructive. A classification of these measurement techniques, with some examples under each type, is presented in Figure 7 [21].

### 4.1. Destructive and Semi-Destructive Methods

Destructive and semi-destructive measurement techniques destroy the specimen part, to release the residual stress in the sample. Destructive techniques are also defined as stress-relaxing methods. The deformation (strain) created by the stress-relaxation when the sample is destroyed is measured, and the measured strain is utilized to calculate the residual stress of the sample based on the general principle of Hooke’s law. Therefore, the accuracy of this method depends on the accuracy of the strain measurement. Despite the harm they do to the specimens, making them appear less desirable than non-destructive procedures, mechanical release methods are frequently the best option, due to their adaptability and dependability. Non-destructive methods for measuring residual stress in metals are considered relatively expensive compared to destructive methods.

**Figure 8:**
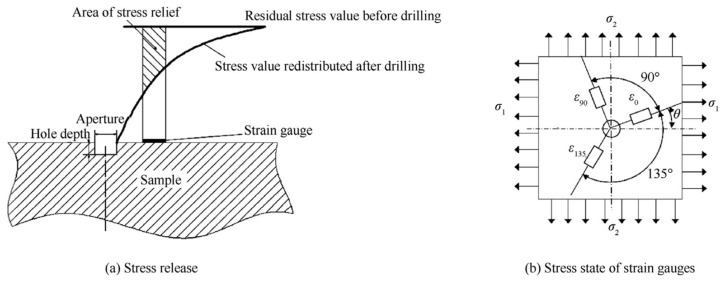
Illustration of the strain gauge hole-drilling method for measurement of RS [45] (open access).

Semi-destructive methods involve fewer modifications in the properties of the specimen compared to destructive methods; hence, they are an intermediary case. The majority of destructive measurement techniques that are frequently applied to calculate residual stress involve hole drilling, ring core, deep-hole drilling, sectioning, and contour techniques. Among the different types of destructive measurement techniques, hole drilling is the most commonly used. In the hole drilling process, a small hole, with typical dimensions of 1.8 mm in diameter and 2 mm in depth, is drilled at the location where the residual stress of the specimen is to be measured. Strain gauges are installed on the surface of the specimen prior to the drilling process. When the material is removed via the orbital drilling process, the specimen undergoes strain relaxation, and the amount of strain is noted using a strain gauge rosette. The measured strain values are used to calculate the residual stress [40]. The hole drilling method was used in measuring the machining residual stresses for turning and grinding [41,42,43] and validated using numerical simulations [44]. A schematic diagram of the strain gauge hole-drilling method is presented in Figure 8 [45].

### 4.2. Non-Destructive Methods

**Figure 9:**
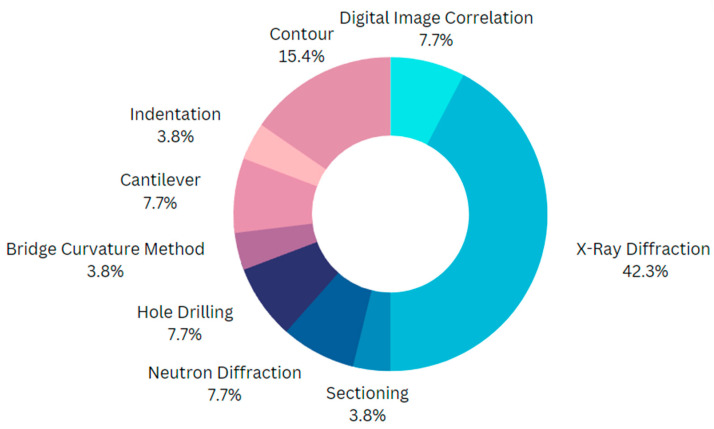
Quantification of the various measurement techniques used by researchers.

Destructive techniques are not viable for parts that are fabricated for application purposes, because the process induces damage to the parts, degrading the integrity and quality of the parts. Therefore, non-destructive techniques are particularly important for expensive specimens, because they do not alter the part integrity significantly. X-ray diffraction, neutron diffraction, the Barkhausen noise method, ultrasonic method, and thermoelastic method are the most commonly applied non-destructive techniques for measuring RS. A survey of 42 papers related to residual stress in AM showed the most frequently used measurement techniques by researchers, and this is presented in Figure 9. It is evident that X-Ray diffraction has been used more frequently by scientists, because of the non-destructive nature of the technique, which preserves the part integrity.

**Figure 10:**
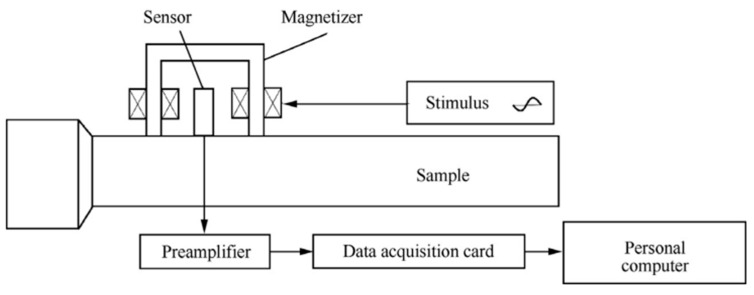
Schematic principle of the BN and MAE detection system [45] (open access).

In non-destructive methods, the crystal lattice strain is assessed using non-destructive techniques (NDT), such as neutron diffraction and XRD. After that, assuming linear elastic deformation of the crystal lattice, the RS is determined using elastic constants. A number of material attributes, including tensile modulus, fracture toughness, microstructure, and flaws, can be revealed using NDT techniques. A schematic diagram of the Barkhauser noise method is presented in Figure 10 [45].

**Figure 11:**
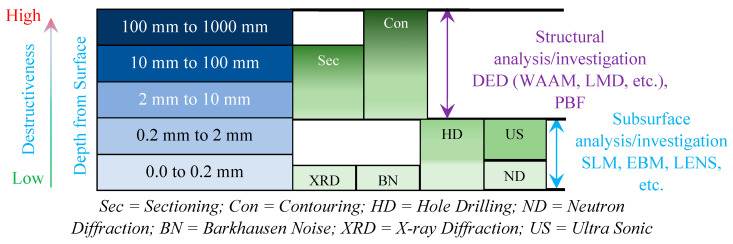
Residual stresses measurement methods for surface and structurally analyzing AM parts.

Although NDT techniques are quick, flexible, and repeatable with high accuracy, there are some limitations associated with NDT methods. Only certain crystalline materials can be measured using X-ray diffraction, depending on how well the surface has been prepared. Only magnetic materials may be tested using magnetic methods, based on how the stress–magnetization curve relationship changes during ferromagnetic saturation. Additionally, ultrasonic testing is suited for depth-varying residual stress measurements and is straightforward in theory, while there are still some challenges to be solved, such as differentiating sound velocity changes brought about by material defects or stress [46]. Table 1 gives a comparison of the different measurement techniques, with respect to the applications of the technique, penetration depth, spatial resolution, advantages, and disadvantages. Figure 11 shows a visual representation of the measurement methods for measuring residual stress when analyzing the surface and sub-surface structures of AM parts.

## 5. Effects of Process Parameters on Residual Stress

The temperature gradient mechanism (TGM) model and the cool-down phase model provide a simplified explanation of how residual stress is formed in metals; however, AM processes are very complex in nature, and the formation of residual stress is, not only affected by the unique thermal cycle of the AM process, but also by the various process parameters. The characterization of residual stress in the AM of metals depends on numerous factors, including beam/laser parameters (power, energy density, and scan speed), process conditions and geometry (layer thickness, part geometry, and powder bed pre-heating), and scan strategy (path optimization, exposure, and re-/pre-melt scan). Table 2 summarizes the effects of varying process parameters on the development of residual stress in metal additive manufacturing.

**Figure 12:**
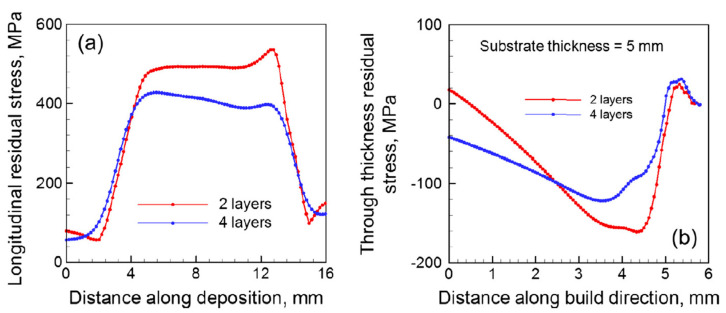
(**a**) Longitudinal residual stress distributions and (**b**) through-thickness residual stress distributions of an IN 718 deposit for process parameters with 2 layers vs. 4 layers [61] (with kind permission from Elsevier).

Researchers have conducted various studies to outline the relationship between processing parameter control and the formation of residual stress. T. Mukherjee et al. [61] investigated the effects of varying layer thickness and beam energy on the formation of residual stress in Inconel 718 components and Ti-6Al-4V parts. In this study, transient heat transfer and a fluid flow model were utilized to outline the temperature field modeling of residual stress and distortion in specimens. The results showed that decreasing the layer thickness reduced the residual stresses in parts by about 20–30%. Under an identical laser power and scanning speed, the volumetric heat flux intensity and the peak temperature were increased for lower layer thickness specimens. Consequently, the total deposition time was higher for lower layer thickness specimens. Although, a longer exposure time and high peak temperature resulted in distortion of the specimens, the residual stress was reduced in the deposition, due to the presence of deformation [62]. Figure 12 [61] shows the distribution of longitudinal residual stresses and the through-thickness residual stresses for process parameters using 2- and 4-layer disposition when building a 0.8 mm wall. When the beam energy of the heat input was doubled, while keeping the other process variables constant, the maximum longitudinal residual stress reduced by 20%; however, the strain in the specimen increased by 2.5%. The distortion created by doubling the heat input significantly affected the part geometry and dimensional accuracy. Therefore, the trade-off of distortion and residual stresses should be considered, depending on the application and use of the parts.

Yu Zhan et al. [64] measured the residual stress in laser additive manufacturing of TC4 by varying the process parameters of scanning speed, laser power, and powder feeding rate. They explained that the LAM specimens had a low level of residual stress domain, and the residual stress distribution perpendicular to the direction of laser scanning was higher compared to the parallel direction. The results showed that by increasing the laser power, the residual stresses in the specimens also increased. The uneven temperature distribution of the melt pool due to high-energy sources resulted in stress accumulation in specimen; hence, a higher laser energy increased the residual stress in the specimen. The residual stress in the specimens was reduced when a high scanning speed and high powder feeding rate was used as process variables. A high scanning speed and high powder feed rate lowered the energy input in the melt pool; hence, an even temperature distribution was acquired in the substrate, resulting in a reduction in residual stress.

In addition to varying scan speed and beam power density, G. Vastola et al. [63] investigated the effect of beam size and bed pre-heating temperature on the residual stress of Ti-6Al-4V specimens. The results obtained when varying the scan speed and beam power were similar to the results published by Yu Zhan et al. [64]. When the beam power was increased by 20%, the heat-affected zone size increased by 15%. Similarly, when a low scanning speed was used, the heat-affected zone size was extended, resulting in high residual stresses. However, they also mentioned that the highest value residual stresses were unaffected by the scanning speed. In regards to the beam size, higher stresses in a smaller heat affect zone were obtained when a small beam size was used. In particular, the depth profile of the residual stresses was affected by the relative beam size. A larger beam size produced uniform stress in a wider heat-affected zone. When the bed pre- heating temperature was increased, the residual stresses in the specimen significantly reduced. The experiment result showed a decrease in residual stress by 20% when the powder pre-heating temperature was raised by 50 °C. The temperature distribution is more even when the powder pre-heating temperature is increased, resulting in lower thermal gradients in the specimen, thus, the residual stress is decreased.

**Figure 13:**
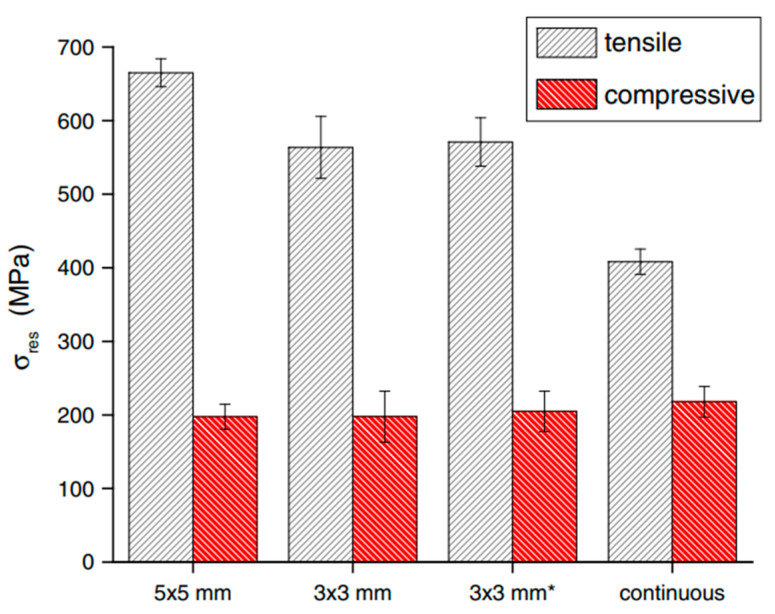
Residual stresses in vertically built AM parts measured via DIC/sectioning; * 0.6 mm x-y offset between layers, as opposed to the typical 1 mm [67]. (with kind permission from Springer Nature).

Amanda et al. [67] investigated the effects of scan island size, island-to-wall rotation, and applied energy per unit length (laser power/speed) on the residual stresses induced in 316 L stainless steel. The effect of a varying scan strategy on the induced residual stress of the specimen was noted by varying the island size and island rotation. When the island size was reduced from 5 × 5 mm to 3 × 3 mm, the tensile residual stress decreased. This was because the curling angle reduced in the bridge-like specimens when the scan vector length was reduced, resulting in lower residual stresses for smaller scan vectors [68]. The effect of varying island sizes on the residual stresses in vertically built AM parts is presented in Figure 13 [67].

In-plane strain components were affected by varying the island orientation/power/speed; however, the axial strain was unaffected. When the build parameters were set to 250 W and 1800 mm/s, the tensile residual stress the in y-direction was significantly reduced, compared to specimens with build parameters of 400 W and 1800 mm/s. This phenomenon was attributed to an increase in island rotation from 0 to 45 degrees, rather than attributing it to an increase in laser power. The use of 45 degree off-axis scanning reduced the residual stresses, which resulted in a useful misalignment between the largest part dimension and the maximum thermal stresses (scan direction). The results of the interaction between the laser power and speed showed that the amount of residual tension caused deflection during sectioning decreased as the energy per unit length increased.

**Figure 14:**
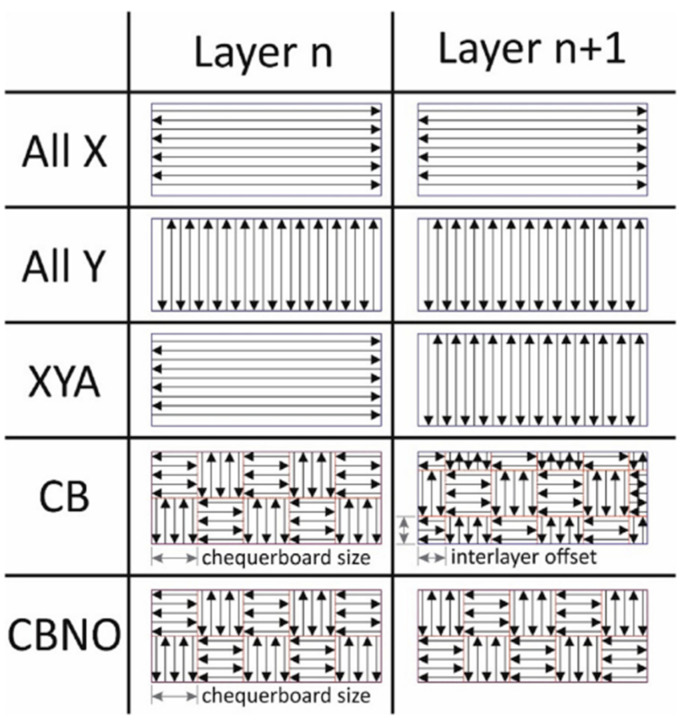
Schematic of scanning strategies [65] (open access).

In a similar study conducted by J. Robinson et al. [65], the researchers investigated the effect of different scan strategies on the residual stress in laser PBF. A schematic of the various scanning strategies used in the study is presented in Figure 14 [65].

The results obtained in the study showed that the all Y scanning strategy showed lower stress values in the measurement direction compared to all X. This was attributed to the fact that the residual stress was primarily in the direction parallel to the scanning direction [23,26,66]. The lowest value of residual stress (maximum uniform distribution) was obtained using an XY alternating strategy for a multi-directional scanning strategy. The chequerboard scanning strategy was less effective compared to the XY alternating strategy. However, when the chequerboard size was increased, a lower value of residual stress was noted, comparable to the XY alternating strategy.

Karan et al. [69] investigated the effects of substrate thickness, interlayer temperature, deposit height/number of layers, substrate size, and geometry on the induced residual stresses of aluminum alloy 5183 manufactured using the WAAM process. The results showed that the samples with a thicker substrate (20 mm) developed maximum tensile stress near the top end of the deposit, despite a change in interlayer temperature or heat input. For thinner substrates, highest tensile residual stress was developed at the junction of the deposit and substrate. The peak value of the residual tensile stress was unchanged, despite the change in deposit height, interlayer temperature, heat input, and substrate dimension. The residual stress for samples with 50 °C interlayer temperature showed 20% increment as compared to sample with 100 °C interlayer temperature. For samples with a lower deposit height, the stresses were tensile in nature; in contrast to samples with a taller deposit height, where the stresses were compressive.

**Figure 15:**
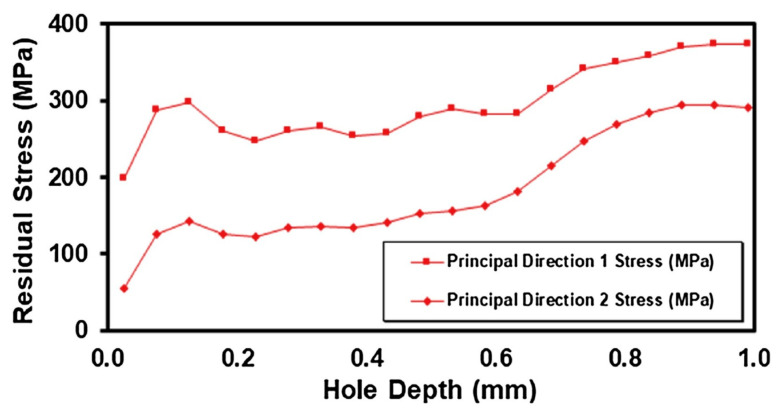
Principal RS distribution on the top surface of a 12.7 mm height deposit based on the hole drilling method [70] (with kind permission from Elsevier).

N.C. Levkulich et al. [70] investigated the effect of build height and substrate overhanging on the development of RS and distortion of Ti-6Al-4V using a laser powder bed fusion technique. Samples were prepared based on different deposit heights of 0 mm, 1.6 mm, 12.7 mm, and 25.4 mm, whereas the length and width of the sample were kept constant. The measurement of RS was performed using the XDR technique and validated using the hole drilling method. The results from both the XDR and hole drilling experiments showed that the RS on the top surface of the deposit decreased as the build height increased. This phenomenon was explained by the presence of a thermal gradient between the newly added layer and the material below it. Initially, each substrate was at room temperature, before the PBF processing. When a new layer was added, a large thermal gradient was developed between the newly added layer and the material below it, resulting in high non-uniform shrinkage during solidification. As the layers kept on increasing, the thermal gradient between the newly added layer and the layer below it decreased, resulting in less non-uniform shrinkage of the deposit during solidification. In contrast to the results for the top surface of the deposits, the RS on the bottom surface of the substrate increased as built height increased. This was due to the contraction force imposed by the newly added layer to the layer below it during the solidification process. The distribution of residual stress measured by hole drilling on the top surface of the 12.7 mm height deposit is presented in Figure 15 [70].

## 6. Numerical Modelling Approach and Validation for Residual Stress

A dependable and well-tested distortion and the residual stress prediction model would be of considerable use for improving the process optimization of AM technologies. It could save time and money compared to experimental methodologies and help researchers gain insight into the development and parameter dependency of residual stress in the additive manufacturing process [37,71,72]. The modelling of residual stresses developed during different additive manufacturing processes involves two analyses: thermal analysis and mechanical analysis. The temperature distribution in the build deposit is determined by thermal analysis, whereas the distortions in the build deposit due to the various thermal loads are determined by the mechanical analysis. These two analyses can be coupled or uncoupled. If a thermal analysis is coupled with a mechanical analysis, the mechanical analysis is subsequently performed at every unit step of the thermal analysis, in order to account for the heat generation due to deformations. The thermal and mechanical history of the deposit at every unit step can be determined both explicitly and implicitly. AM is a highly non-linear process; therefore, an explicit approach is useful. The computational time, however, for AM simulations may take longer, because the convergence requires a very small unit step size. Adaptive time steps can be utilized to increase the efficiency of the simulation process. An adaptive time step utilizes small time steps for a system that is changing rapidly, whereas a larger time step is applied for slower processes. Such a computational method was utilized by Zohdi et al. [73], in order to reduce the computational time.

A simulated model of the AM process can be at the micro-level or macro-level. The interaction between the heat source and feedstock is included in micro-scale models of AM processes. While macro-scale models employ the dimensions of the heat-affected zone and the thermal cycle to compute the residual stress in components, micro-scale models provide information about the size of the melt pool, temperature distribution, and material consolidation quality. Usually, the residual stress modelling of AM is carried out at macro scale, with the assumption of heat distribution from a source. However, for accurate measurement of RS, it is recommended to model the interaction of the heat source and feed-stock at the micro-level and to model the interaction of fluid flow to develop the temperature distribution of the system. The obtained temperature distribution of the system can be utilized using macro-modeling, to reveal the residual stress and distortion present in the system [74]. Once the modelling approach and time integration are established, energy conservation and heat transfer laws are employed to determine the motion of heat flow from the heat source (laser/beam) to the sink. Heat dissipation from the system to the surrounding is accounted for using convection and radiation from the free surfaces and conduction through the material.

**Figure 16:**
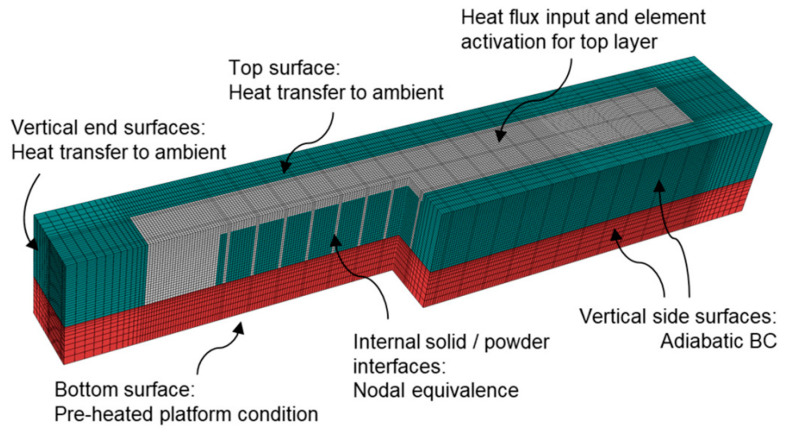
SLM model set-up with build platform using a unit cell approach with adiabatic surfaces [75] (open access).

Xu Song et al. [75] utilized a finite element modelling approach using the ABAQUAS AM module to simulate the LDED and SLM processes. The researchers aimed to optimize the AM process parameters using the finite element approach, to reduce the residual stresses and distortions that are commonly encountered in AM processes. To achieve process simulation of complicated 3D parts, this module offers an automated interface that enables the user to describe data such as the tool path, element activation, build environment, and heat input as a function of position and time. The simulated data for the thin-wall structure in the LDED process and overhanging structure in the SLM process were experimentally verified using FIB-DIC and XDR measurement. The set-up of the SLM model with the build platform using a unit cell approach is presented in Figure 16 [75].

The local temperature profile was successfully predicted using the ABAQUS AM module and validated using fine mesh approaches with Goldak heating and coarser mesh resolutions. The results showed that the simulation time could be reduced if the time step resolution was increased; however, this could negatively affect the prediction of the local peak temperature profile.

Jinqiang et al. [76] utilized an analytical modeling methodology to predict the distortion and residual stress in the PBMAM process. The applied model utilized four different modeling approaches: thermal modeling, residual stress modeling, thermal stress modeling, and distortion modeling. Since this modeling approach did not utilize iteration-based calculation, the computational efficiency was greatly improved, reducing the computational time. The temperature profile was predicted utilizing a moving-point heat source and sink solution. The thermal load, hydrostatic pressure, and heat sink were utilized to calculate the temperature using the thermal stress model. The obtained temperature was utilized to calculate the thermal stress. Elastoplastic relaxation was used to calculate the residual stress. Finally, the assessment of distortion was predicted using the surface displacement model, utilizing the calculated residual stress and residual strain. The obtained analytical calculation was verified experimentally by measuring the distortion for the twin-cantilever beam using a coordinate measurement machine (CMM). The analytical approach showed a close agreement with the experimental data.

**Figure 17:**
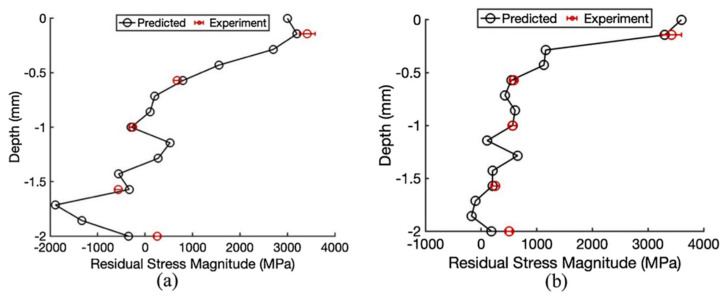
Predicted RS of IN718 with the laser power of 485 W and scan speed of 40 mm/s (**a**) along scan direction; (**b**) along the build direction [77] (with kind permission from Elsevier).

Elham et al. [77] utilized an analytical model to predict the residual stress in metal by considering the microstructure evolution. A transient moving point heat source was used to predict the thermal behavior. The yield surface was predicted using a modified-cook flow stress model. The effect of grain size was incorporated using the Hall–Petch equation. The Johnson–Mehl–Avrami–Kolmogorov (JMAK) model was used to predict the resultant grain size and the dynamic recrystallization. The rapid solidification of the grain size was incorporated using a grain refinement model. Finally, the kinematic hardening behavior of the metal and incremental plasticity were used to predict the residual stress buildup. X-ray diffraction experimentally measured the residual stress, and the analytical results were compared. The results showed a good agreement of the analytical calculation with the experimental results. The maximum calculation error was found to be around 21%. A comparison of residual stress obtained from the analytical results and experimental results during DMD of IN718 using a laser power of 485 W and scan speed of 40 mm/s is shown in Figure 17 [77].

Xinran et al. [78] developed a numerical model in a COMSOL Multiphysics environment, to predict the thermal behavior and residual stress development in the DMLS process for a titanium alloy by considering the temperature-dependent material properties. The model was able to predict the thermal history, melt pool size, and residual stress of a single layer, as well as multiples layers, when the base temperature and laser power was varied. According to the outcomes of the numerical simulation, the melting and solidifying processes took place at a single site in a matter of milliseconds (approximately 1 ms). Therefore, the planar heating model provides the prediction of small-scale local phenomena with a significantly lower computational cost and reasonable accuracy. The melt pool size was simulated based on uniform laser heating, and the simulated melt pool size was slightly smaller in depth and slight wider in diameter compared to the actual melt pool size. The residual stress analysis showed that the vertical normal stress was significantly smaller in magnitude than the horizontal normal stress, and the shear stresses were hardly noticeable. When the base temperature increased, the residual stress’s average value decreased. The addition of plasticity resulted in lower stress values and higher strain values compared to the pure elastic behavior. Guangjie et al. [79] developed a thermal-elastic-plastic finite element method to numerically simulate the thermal field, stress field, and distortion during the WAAM process. The distribution of the temperature field, stress field, and distortion was studied based on varying the substrate thickness and inter-pass temperature. The created finite element method’s dependability was confirmed using the calculated results for the angular deformation of the substrate, which were in good agreement with the experimental findings. Heng et al. [80] modeled the residual stress and distortion in the MAM process using a thermo-mechanical finite element method. The simulation was successful in explaining the temperature distribution, thermal stress field, and distortion in parts. Using a laser displacement sensor, experimental data for the mechanical response were acquired and compared with numerical modeling data for validation. The simulated and experimental results were in good agreement.

## 7. Methods for Mitigating Residual Stress

Apart from the process parameters of the control and pre-heating of the substrate or feedstock material, several post-processing techniques can be applied to mitigate the residual stress present in the MAM process. The most commonly applied post-processing techniques for metal additive manufacturing include post-heat treatment, laser shock peening (LSP), shot peening (SP), ultrasonic shot peening (USP), ultrasonic impact treatment (UIT), inter-pass rolling, machining, laser polishing, and chemical and electrochemical polishing. Among the mentioned post-processing techniques, heat treatment and the LSP process have been studied the most for reducing the RS in the MAM process.

### 7.1. Heat Treatment

**Figure 18:**
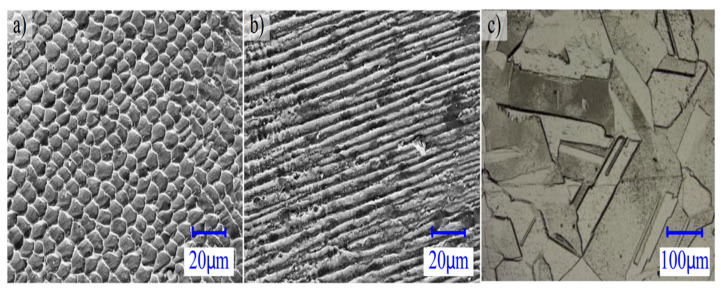
Microstructure of samples with different post-heating conditions: (**a**) as-built, (**b**) heat treated at 400 °C for 2 h, and (**c**) heat treated at 1150 °C for 2 h [81]. (With kind permission from Springer Nature).

The fatigue performance of parts can be improved using stress relief heat treatment, because this helps to decrease the tensile residual stresses. Through a carefully regulated heat treatment process, the brittle microstructures in as-built AM metals can be made stable. Heat treatment can alter the microstructure of metals, improving their ductility and strength. Pengfei et al. [81] investigated the effect of post-heat treatment on the residual stress of 316 L stainless steel fabricated using the DLD process. The results showed that the tensile residual stress of the specimen decreased significantly when a heat treatment was applied at 400 °C for 2 h. The tensile residual stress was reduced to 356.29 MPa (53.7% reduction) for samples that were heat treated at 400 °C for 2 h compared to 769.27 MPa for untreated samples. At the same temperature, the value of yield strength and UTS were unchanged, although the elongation was increased to 40.2% from 26.48%. A continuous increase in the heat treatment temperature resulted in a decrease in yield strength and UTS for the specimen. The microstructural evolution of the samples at different post-heating conditions is presented in Figure 18 [81].

Yeong Kim et al. [82] investigated the effect of heat treatment on the residual stress present in aluminum alloy fabricated using the PBF process. The fabricated samples were heat treated in a vacuum at a temperature of 480 °C or 550 °C for 6 h, then allowed to cool down naturally in a furnace. The heat treatment process changed the tensile residual stress (46–188 MPa) of the untreated sample to compressive residual stress (28–51 MPa), with a significant reduction in the magnitude of residual stress. Xiaoqing et al. [83] applied a stress-relieving heat treatment to Inconel 718 fabricated using the PBF process. The heat treatment process resulted in the development of a homogeneous microstructure in the specimen, increasing its microhardness by 19%. Similarly, the maximum absolute residual stress was reduced to 321 MPa from 378.4 MPa when stress relief HT was applied. Richard et al. [84] investigated the effect of heat treatment on the residual stress of 316 L stainless steel fabricated using the PBF technique. A heat treatment was carried out at 700 °C for 2 h. The relaxation effect of the heat treatment process preserved the unique microstructure of the AM process. The peak value of residual stress was reduced by 10% in vertically built samples and by 40% in horizontally built samples after the heat treatment process.

### 7.2. Laser Shock Peening (LSP)

**Figure 19:**
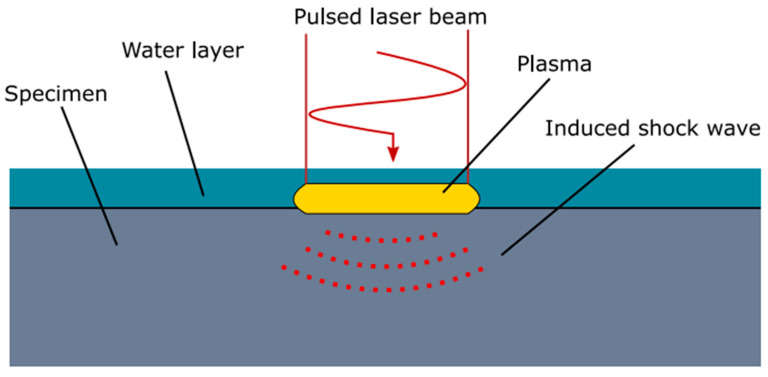
Schematic of Laser Shock Peening (LSP) process. The materials close to the surface are vaporized by the shock wave induced by the laser pulse, creating a rapidly expanding plasma. The shock wave will induce local plastic deformation, resulting in compressive residual stresses beneath the top surface [87] (Open access).

Laser shock peening (LSP) is a surface treatment process in which compressive stresses are developed in the specimen. The LSP process is beneficial, because it modifies the residual stress profile of AM components [85,86]. The LSP process is similar to shot peening (SP) and ultrasonic shot peening (USP); however, it utilizes the application of a laser. A schematic diagram of laser shock peening (LSP) is presented in Figure 19 [87]. Veronica et al. [88] investigated the effect of laser shock peening on the residual stress of AM stainless steel parts. The results showed that LSP had much less effect in the XZ-build direction; however, LPS produced a reduction in tensile back-stress in the XY-built direction of 247.61 MPa. This result was attributed to the alignment of the peening axis to the built direction, resulting in compressive hardening of the surface material and reducing the tensile back stress. Back-stress reduction in the XZ samples was negated by tensile hardening, because the texture of the XZ-built samples was softer with tensile loading in the pulling direction than that of the XY-built samples.

**Figure 20:**
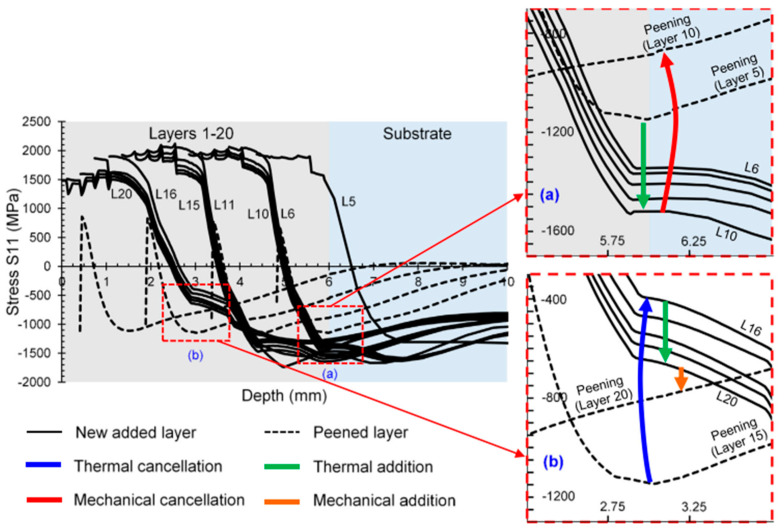
Stress profile evolution in hybrid AM process with laser peening on every five layers; (**a**) the stress profile after adding 10 layers and applying LP on the 5th and 10th layer; (**b**) the stress profile after adding 20 layers and applying LP on the 15th and 20th layer [89]. (Open access).

Guru et al. [89] investigated the thermal and mechanical cancellation of residual stress in a hybrid AM process using laser peening. The study aimed to understand the effect of mechanical and thermal cancellation induced by cyclically coupling printing and peening on spatial and temporal residual stress development. The finite element modeling of the hybrid AM process showed the development of compressive residual stress in the workpiece, induced by the layer-by-layer peening process. The depth of compressive residual stress was increased when the frequency of the layer peening was reduced to 5 from 20. The depth of compressive residual stress was unaffected when peening was performed more frequently, as compared to peening after every five layers. The single peening mode resulted in a compressive residual stress value of ±100 MPa for all peening frequencies. Thermal cancellation, thermal addition, and mechanical cancellation were visible in the stress profile progression in the model with a layer peening frequency of ten. When peening every five layers, thermal addition and mechanical cancellation were seen in layers 5 and 10. Peening each layer revealed no mechanical cancellation. The evolution of the stress profile of hybrid-AM model with laser peening every 5 layers is presented in Figure 20 [89].

In a similar study conducted by Rujian et al. [90], researchers combined the WAAM process with laser shock peening, to create a hybrid AM process. The hybrid AM process refined the microstructure of the specimen, improving its mechanical performance and modifying the stress state. The average grain size was reduced to 46.7 μm from 59.7 μm when peening was applied. The tensile residual stresses were changed into compressive residual stresses with a maximum magnitude of 100 MPa. The yield strength of the specimen was increased by 72%.

### 7.3. Rolling

Rolling is a post-processing technique that is applied to eliminate residual stress and distortion present in AM metal components. Rolling can be performed in normal ambient conditions (cold rolling), as well as under the influence of high temperature and pressure. Rolling introduces plastic deformation in AM metal parts, resulting in lower porosity and microstructural anisotropy. However, rolling is limited to parts that have simple geometries, such as straight walls or flat surfaces. For parts with complex geometries, the rolling process can be complicated and may require specific flexible tools [91].

F. Martina et al. [92] investigated the effect of rolling on as-deposited Ti-6Al-4V components manufactured using a wire + arc technique. The residual stress in the as-deposited components was measured using the contour method and was verified using the neutron diffraction technique. The results showed that the tensile residual stress (500 MPa) presented in the unrolled sample was successfully reduced to 200 MPa by inter-pass rolling. In addition to reducing the tensile RS at the interface of the substrate and the linear deposit, inter-pass rolling resulted in high compressive stresses near the top surface. However, geometrical modifications were noticed in AM components, resulting in increased wall widths and reduced layer heights. While rolling did not completely eliminate the distortion presented in components, the overall distortion was reduced to half compared to unrolled components. In a similar study conducted by Hönnige et al. [93], researchers investigated the effects of inter-pass rolling on the residual stress and distortion control of aluminum components manufactured using wire + arc techniques. The results showed that post-deposition side-rolling was very effective in reducing the residual stress and distortion in aluminum components. Vertical inter-pass rolling was found to be effective in completely eliminating the distortion present in the aluminum components, unlike the Ti-6Al-4V components mentioned in the previous study [92]. In addition, the yield strength and tensile strength of the rolled components were increased, due to the increased hardness caused by work hardening during inter-pass rolling.

Luis et al. [94] applied finite element analysis to simulate two different rolling methods: rolling the weld bead directly with a single roller, and rolling with a dual flat roller. The simulation results showed that both rolling techniques were able to induce compressive stress in the weld region. The induced compressive stress was increased when the rolling load was increased. The longitudinal RS was reduced by rolling the weldments beside the weld beads. The reduction was highly dependent on the position of the roller in relation to the weld bead. When the roller “pinched” the weld profile, higher reductions were obtained. Paul et al. [95] investigated the effects of high-pressure rolling on parts manufactured with the WAAM process. Researchers investigated two different rollers—a profiled roller, and a slotted roller—to identify the effect of the roller profile on the suppression of RS in the WAAM components. The results showed that both rollers were effective in reducing the distortion and surface roughness of the parts; however, the slotted roller proved to be more effective in eliminating distortion and RS compared to the profiled roller. The subsequent reheating of parts after each pass resulted in an additional grain refinement in parts, in addition to a reduction in RS and distortion.

### 7.4. Ultrasonic Impact Treatment (UIT)

**Figure 21:**
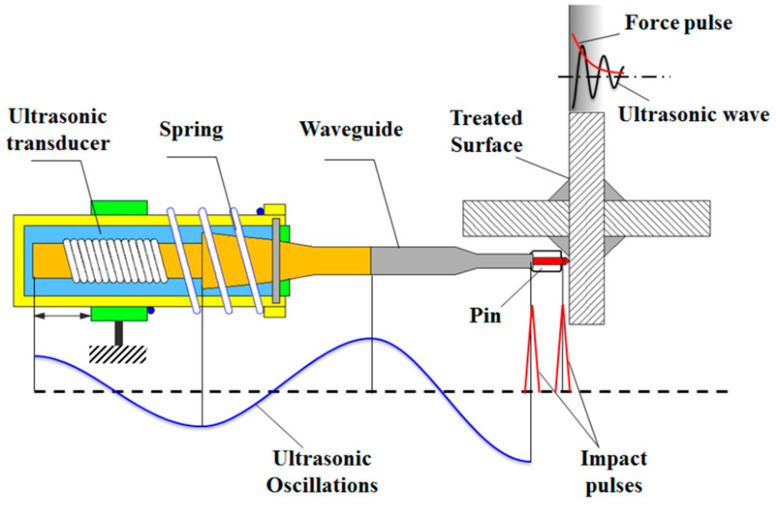
Ultrasonic impact treatment (UIT) process [96] (open access).

Ultrasonic impact treatment (UIT) is a post-weld technique in which a mechanical impact is applied in combination with ultrasonic oscillation in welded joints, to introduce plastic deformation and compressive residual stress in components. A schematic diagram of the UIT process is presented in Figure 21 [96]. UIT is a cold mechanical treatment in which a high-energy source is used to contact the weld surface and introduce beneficial compressive residual stress into components. The mechanical strength of the component is also improved, due to the grain refinement and micro-hardness improvement. The penetration depth of UIT treatment is about 60 m from the top surface; hence, UIT treatments are more effective for shallow depth parts [97].

Jianfei et al. [98] investigated the effects of UIT treatment on the development of RS in the repair of welding joints. The experiment results showed that the residual stress in welding was mainly concentrated in the heat-affected zone, and the magnitude of RS was smaller in regions away from the HAZ. After the UIT treatment, the RS in parts was reduced significantly. The peak and average longitudinal RS were reduced to 66.3 MPa and 7.6 MPa, from 275.9 MPa and 270.8 MPa, respectively. The transverse residual stresses were converted into beneficial compressive stresses after the UIT treatment, for thin specimens, the overall RS was reduced significantly after UIT treatment; however, for thick specimens, the RS in the middle layer was increased after UIT. UIT treatment enabled plastic deformation and energy stability in the parts, resulting in the relaxation of RS.

Lanqing et al. [99] investigated the effect of UIT treatment on 304 L stainless steel welded joints using a numerical simulation method. Finite element modelling was applied to simulate both the welding and the UIT treatment process. The obtained RS from the FE model was validated using experimental data. The results showed that the UIT treatment of welded parts decreased the fatigue growth rate and improved the fatigue life of the welded parts. UIT was successful in introducing compressive residual stress in components up to a depth of 2–3 mm.

## 8. Conclusions

Metal additive manufacturing (MAM) has several advantages over subtractive and traditional manufacturing processes. The ability to fabricate complex shaped geometries with reductions in material waste, energy, cost, and time makes the MAM process a growing industry, and applications have been realized in the medical, automotive, and aerospace fields. However, the sustainable development of MAM technologies is restricted by inherent problems associated with the manufacturing of high-quality parts. The staircase effect and dependency on various process parameters restricts AM technologies from reliably producing high-quality parts, hence its application is limited. Residual stresses in AM metals have been identified as a key factor opposing the production of high-quality parts. Scientific communities have made various attempts to characterize the evolution, mechanism, and development of residual stress in the MAM process. Characterization of residual stress based on varying process parameters is a key necessary step that ensures the reliability of the MAM process.

In this paper, the current state of AM technologies was thoroughly investigated. Several AM technologies were identified, and the problems existing in AM process were outlined. Residual stress was determined as the key factor for reducing the fatigue life and mechanical performance of fabricated AM parts. Measurement techniques for measuring the RS in metal were classified, and the advantages and disadvantages of each measuring technique were established. The characterization of RS based on varying the process parameters was identified. The proper control of these process parameters is essential for in situ control of residual stress. Similarly, numerical simulation was identified as an important technique for measuring and predicting the evolution of RS in metal AM with varying process parameters.

## 9. Recommendations for Future Research on Residual Stress in AM

Post-processing of metal additively manufactured parts is recommended to improve the surface integrity, including the surface roughness, sub surface residual stresses, and microstructure. Post-processing methods to improve surface integrity are well established in conventional manufacturing processes, such as machining, grinding, chemical etching, etc. Research on using conventional material removal processes to improve metal additive manufactured part surface integrity is essential for improved part quality and life. However, machining may induce additional residual stresses on metal AM parts. As a result, specific post-processing methods targeted towards the improvement of residual stress need to be developed.

Sustainable post-processing and sustainable machining of metal AM parts have become important areas of research in recent years. Sustainable post-processing using MQL and cryogenic coolant was found to provide an improved surface finish and reduced burr formation [100,101]. However, it will be interesting to investigate if sustainable machining influences (either positively or negatively) the state of residual stress in machined or post-processed parts. Table 3 summarizes the effects of various post-processing techniques that have been studied to improve the formation of residual stress in AM metals.

Residual stress generation in metal AM processes, especially in AM processes associated with melting and solidification, is an inevitable phenomenon. Therefore, our understanding of the mechanism of residual stress formation is of prime importance. Physics-based models for understanding the residual stress generation and the influence of post-processing can be used to better understand and optimize the AM process parameters.

Most of the current techniques for the measurement of residual stress (i.e., XRD methods or hole drilling method) require expensive equipment and/or technical expertise. In many cases, these two methods are difficult to apply in an industrial setting. Future research should focus on the design and development of innovative experimental techniques to measure the residual stress of metal AM parts in a simple and inexpensive way. A dependable and well-tested distortion and residual stress prediction model would be of considerable use for improving the process optimization of AM technologies. This could save time and money compared to experimental methodologies and help researchers gain insight into the development and parameter dependency of residual stress in the additive manufacturing process.

It is now obvious that the same complex metal 3D part can be manufactured using different metal 3D printing processes. Metal 3D printing processes not only vary in terms of process mechanisms, capabilities, and part qualities, but also vary in the amount and type of residual stress generated during the 3D printing processes. It is important to carry out comparative analysis of the residual stress generated by different AM processes when making a single part and to consider that analysis in the selection of a particular metal AM process.

Designs for residual stress in metal AM should be an area of focus of future research. Currently, design for additive manufacturing is an important area of research. As the additive manufacturing field grows, individual design criteria become an important topic to consider. Residual stress remains one of the major challenges in metal AM processes that has to be considered when designing a part. Therefore, it is expected that design for residual stress in metal AM will become an important research area for future researchers. Although, methods for mitigating residual stress were discussed at the end of this review paper, many opportunities remain to work on this area of research. Currently, the majority of researches are focusing on the measurement of residual stress in already printed parts and applying post-processing techniques for relieving the residual stress in a 3D printed part. Post-processing methods are particularly important, because these processes further enhance the qualities and properties of components, which is impossible to achieve via 3D printing alone. However, future research should also consider relieving residual stress in situ, as the part is built layer-by-layer.

The residual stress that remains in the part also influence the post-process machining performance of metal AM parts. The challenges in maintaining the dimensions and part quality after post-processing due to residual stress has been discussed in various research studies. However, the influence of residual stress on the machinability aspects of metal AM parts has not been studied in depth in the literature. For example, the effects of residual stress on cutting forces, tool wear, surface integrity, and burr formation when post-processing metal AM parts need further attention. The tool wear mechanisms for the machining of traditional cast or forged metal will be different from the machining of AM metal parts, and residual stress may have a significant influence on that difference. However, the basic knowledge of tool wear mechanisms [111] and burr formation mechanisms [112] may be applicable to metal AM parts to some extent, and future research should answer this research question.

In addition, post-process machining of 3D printed polymer or metal parts imposes other challenges, such as delamination, decohesion, and debonding of layers [113]. Numerical modeling can explore some of those phenomena by revealing the underlying physics behind them. Residual stress generation during metal AM can further contribute to these phenomena. Therefore, future research should focus on investigating the effects of residual stress on the delamination, decohesion, and debonding phenomena commonly seen during post-process machining of metal AM parts.

In recent years, various thermal material removal processes (i.e., EDM, laser cutting etc.) have been used for the post-processing of metal AM parts [114]. EDM and micro-EDM with low discharge energy levels are typically used to support structure removal and improve the surface finish of AM parts. However, EDM may induce additional thermal residual stress while cutting or finishing metal AM parts. On some occasions, the release of residual stress from the metal AM parts during support structure removal causes thermal distortion. Future research should focus on the design and development of the pulse generator for EDM, as the design of pulse generator was reported to have a significant influence on micro-EDM performance [115]. Future research also should focus on the design and development of innovative pulse generators and/or energy sources capable of supplying very small energy levels, which could minimize the thermal residual stress and resulting distortion and improve surface finish and dimensional accuracy.

The application of machine learning (ML) and artificial intelligence (AI) to optimizing the process performance of additive manufacturing has received much attention in recent years and will remain a major focus of manufacturing research in the next decade [116]. As a result, researchers working on mitigating or managing residual stress in metal AM should consider applying ML and AI techniques to monitor and control the generation of residual stress and to optimize AM process parameters, printing process, and post-processing techniques, to minimize and control the amount of residual stress generated during metal AM.

## Figures and Tables

**Table 1:** Comparison of different measurement techniques based on penetration depth, spatial resolution, applications, advantages, and disadvantages.

Measurement Technique	Penetration Depth	Spatial Resolution	Applications	Advantages	Disadvantages	References
Hole-drilling	1.8 mm to 2.0 mm deep	-	Wide range of elastic and isotropic materials, used in machining surface integrity research	Simple experimental setup, straightforward operation, improved accuracy, relatively accurate	Semi-destructive, limited to strain sensitivity, material needs to be machinable, source of errors includes non-cylindrical hole, stress addition due to machining, and eccentricity	[40,43,47]
Sectioning	2 mm to 100 mm	-	Wide range of materials like Structural carbon steel, aluminum, stainless steel etc.	Fast and economical	Destructive, limited stain resolution	[48,49]
Contour method	2 mm to 1000 mm	-	Application includes quenched and impacted thick plates, cold-expanded holes, aluminum alloys, carbon steels	High spatial resolution, wide range of materials, large samples	Destructive, Additional stresses can be introduced, and data interpretation is required	[50,51,52]
X-ray diffraction	100 μm (Ti) 50 μm (Al)	Transverse: 1 mm Depth: 20 μm	Applicable to materials that are crystalline, fine grained, and produce diffraction at any orientation, used in machining surface integrity research	Non-destructive, high accuracy, can measure both macro and micro-RS	Only applicable for measuring RS near the surface, lab-based system, expensive	[53,54]
Neutron diffraction	0.2 mm–100 mm (Al) 0.2 mm–25 mm (steel) 0.2 mm–17 mm (Ti)	Transverse: 20 μm	Wide range of composites and heterogeneous materials	Can measure both macro and micro-RS, non-destructive, optimal penetration and resolution	Lab-based system, expensive, slow detection speed	[55,56]
Barkhausen noise method	Depends on the permeability of the material, Up to 0.2 mm	-	Application is limited to ferromagnetic materials	Non-destructive, can reveal microstructural parameters and surface RS, fast	Only applicable to ferromagnetic materials, low resolution	[57,58]
Ultrasonic method	0.2–2 mm	0.1–30 m^2^	Application includes the determination of RS and applied stress in real structures	Non-destructive, readily available, fast and cost efficient	Limited resolution, accuracy is compromised by the material and external factors	[59,60]

**Table 2:** Effect of varying process parameters on the formation of residual stress in AM metals.

Process Variable	Effects due to Variation	Remarks	References
Layer thickness	Parts with a thin layer thickness have lower residual stress compared to parts with a thick layer thickness.	Increasing the number of layers from 2 layers to 4 layers for the same height of parts resulted in a 20–30% reduction in RS [61].	[61,62]
Substrate preheating	Preheating the substrate at elevated temperature can significantly reduce the effect of residual stress in AM metals. The distortion phenomenon in a thin substrate can be reduced if clamping is performed along with substrate pre-heating.	Residual stress reduced by 20% when the preheating temperature was increased by 50 °C [63].	[25,26,27,63]
Laser power	Increasing the laser power increases the formation of residual stress in AM metals. In addition, parts are more susceptible to distortion when subjected to a high-power source.	When the beam power was increased by 20%, the heat affected zone size increased by 15% [63].	[63,64]
Scanning speed	High scanning speed reduces the formation of RS.	A high scanning speed lowers the energy input in the melt pool, forming even temperature distribution.	[64]
Scan strategy	The XY alternating scanning strategy is the most effective multi-dimensional scanning strategy for minimizing residual stress.	Continuous deposition is more likely to induce warpage and distortion in parts compared to alternating scanning strategy.	[23,26,65,66]
Powder feeding rate	A high powder feeding rate reduces the formation of RS in AM metals.	A high powder feeding rate lowers the energy input in the melt pool, thus an even temperature distribution is achieved.	[64]
Scan island size	As island size increases, the RS in metals also increases.	Smaller scan vectors result in lower residual stresses because shorter scan vectors reduce the curling angle in bridge-like specimens.	[67,68]

**Table 3:** Summary of the effects of various post-processing techniques for minimizing RS in AM metals.

Post-Processing	Effects	Remarks	References
Post-heat treatment	Heat treatment significantly improves the fatigue life of AM parts, and reduces the formation of RS if appropriate heat treatment temperature is determined. The ductility of the specimens also gets increased.	Heat treatment at 400 °C for 2 h resulted in a 53.7% reduction in residual stress [81]. However, the yield strength and UTS of parts are lowered if heat treatment is carried out at very high temperature.	[81,82,83,84]
Laser shock peening (LSP)	LSP is applied to change the tensile residual stress to compressive residual stress. LSP improves the fatigue life of parts and increases the micro-hardness with grain refinement.	When LSP was applied, the tensile RS was changed to compressive residual stress with magnitude of 100 MPa. The yield strength was improved by 72% [90].	[85,86,87,88,89]
Inter-pass rolling	Improves the grain refinement, and reduces the RS formation.	Inter-pass rolling reduces the anisotropy present in the microstructure due to the induced plastic deformation. Hence, the mechanical properties are also improved.	[102,103]
Hot isostatic pressing (HIP)	HIP treatment induces microstructural changes in AM metals, which reduces the porosity and improves the fatigue life of parts. HIP treatment at elevated temperature can reduces the formation of RS in metals.	HIP treatment can improve the fatigue strength limit of parts by more than 100% compared to non-treated and stress-relieved specimens [104].	[105,106]
Ultrasonic nanocrystal surface modification (UNSM)	UNSM can significantly improves the fatigue performance of metal parts. In this process, parts are subjected to plastic strain, resulting in grain refinement, work hardening, and formation of compressive residual stress.	The UNSM process helps in converting the tensile residual stress to compressive residual stress, which improves the fatigue life of parts.	[107,108]
Shot peening	Shot peening has similar effect to LSP. In shot peening, compressive residual stress is induced, replacing the tensile residual stress.	Compressive residual stress is beneficial for improving the fatigue life of parts.	[109,110]

## Data Availability

Data are available upon request.
